# Surgical Treatment of Paediatric Chronic Rhinosinusitis

**DOI:** 10.3390/jcm8050684

**Published:** 2019-05-15

**Authors:** Sara Torretta, Claudio Guastella, Tullio Ibba, Michele Gaffuri, Lorenzo Pignataro

**Affiliations:** 1 Fondazione IRCCS Ca’ Granda Ospedale Maggiore Policlinico, 20122 Milan, Italy; claudio.guastella@unimi.it (C.G.); tullio.ibba@policlinico.mi.it (T.I.); michele.gaffuri@policlinico.mi.it (M.G.); lorenzo.pignataro@unimi.it (L.P.); 2 Department of Clinical Sciences and Community Health, University of Milan, 20122 Milan, Italy

**Keywords:** rhinosinusitis, children, surgery, adenoidectomy, balloon sinuplasty, endoscopic sinus surgery

## Abstract

Rhinosinusitis (RS) is a common disease in children, significantly affecting their quality of life. Chronic rhinosinusitis (CRS) is frequently linked to other respiratory diseases, including asthma. Children affected by CRS may be candidates for surgery in the case of failure of maximal medical therapy comprising three to six weeks of broad-spectrum systemic antibiotics with adjunctive therapies. Although endoscopic sinus surgery (ESS) is the surgical treatment of choice in adult patients with CRS, different surgical procedures are scheduled for refractory paediatric CRS and include adenoidectomy, paediatric ESS (PESS), and balloon catheter sinuplasty (BCS). The present paper discusses the indications and limitations of each treatment option in children with CRS. Given the amount of current evidence, it is reasonable to suggest that, in young and otherwise healthy children with refractory CRS, an adenoidectomy (eventually combined with BCS) should be offered as the first-line surgical treatment. Nevertheless, this approach may be considered ineffective in some patients who should be candidates for traditional ESS. In older children, those with asthma, or in the case of peculiar conditions, traditional ESS should be considered as the primary treatment.

## 1. Introduction and Anatomic Considerations

Rhinosinusitis (RS) is a common disease in children, significantly affecting the patients’ quality of life [1,2], and disease complications requiring urgent treatment may occur.

RS is defined as the presence of two or more symptoms, one of which should be either nasal blockage/obstruction/congestion or nasal discharge (with or without facial pain/pressure and cough), and either endoscopic signs (i.e., nasal polyps and/or mucopurulent discharge from the middle meatus and/or oedema/mucosal obstruction in the middle meatus) and/or symptomatic changes observed through maxillo-facial computed tomography (i.e., mucosal changes within the ostiomeatal complex and/or sinuses); chronic RS (CRS) occurs in the case of symptom persistence for more than 12 weeks [3]. CRS is a heterogeneous disease in adults, as different phenotypes and endotypes (i.e., biological subtypes defined by corresponding biomarkers and peculiar responsiveness to some medical treatments) may be identified [4].

**Figure 1:**
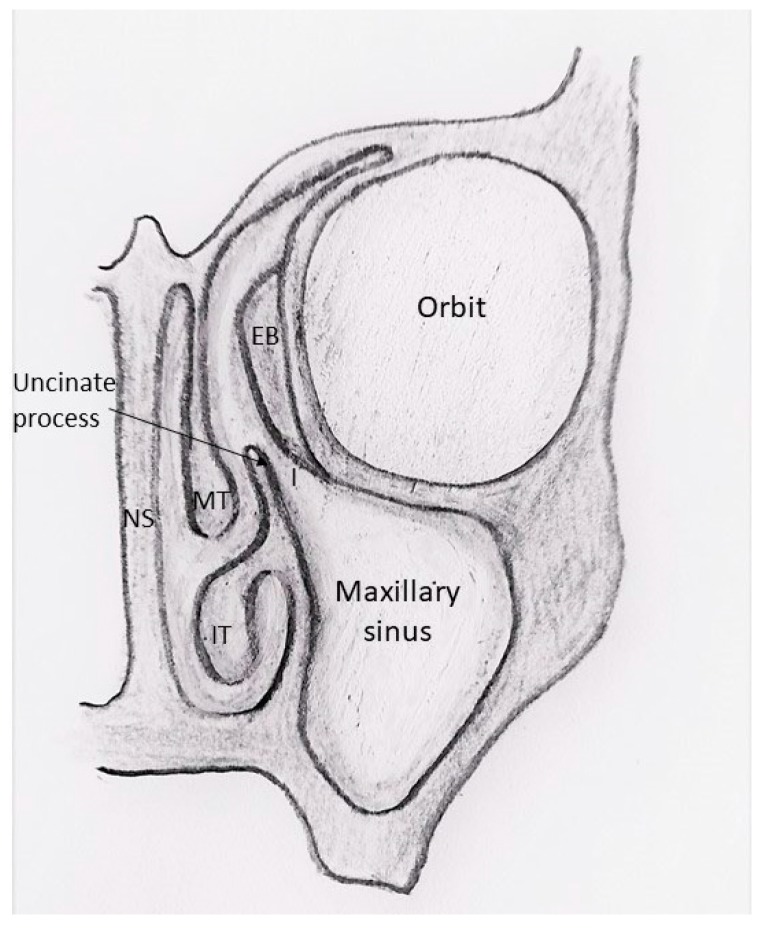
Anatomy of the ostiomeatal complex. MT = middle turbinate; IT = inferior turbinate; NS = nasal septum; I = infundibulum; EB = ethmoid bulla.

Paranasal sinuses are air-filled cavities lined by pseudostratified ciliated epithelium which is in continuation with the nasal cavity. Homestasis of sinonasal drainage is substained by three main factors: the patency of the sinusal obstia, the presence of an adequate and active mucous production, and an effective ciliary function. The mucocicilary clearance transports sinusal secretions through the sinusal obstia into the nasal cavity. The ostiomeatal complex (Figure 1) is an important area of sinusal drainage placed in the middle meatus where secretions from the maxillary, the anterior ethmoid, and the frontal sinuses converge. It is composed by the natural obstium of the maxillary sinus, the infundibulum, the uncinated process, the hiatus semilunaris, and the ethmoid bulla (Figure 1). On the other hand, secretions from the posterior ethmoid and the sphenoid sinuses are directed through the sphenoethmoidal recess, placed in the superior meatus.

Viral upper respiratory tract infections are frequently inciting events, as they induce a transitory ciliostasis, predisposing to bacterial overgrowth. Possible causative factors are allergic and nonallergic rhinitis, airway pollution, smoke exposure, and prolonged nasotracheal tube placement [5]. Moreover, underlying systemic diseases may act as predisposing factors for the development of CRS with or without nasal polyps [6,7]. 

Additionally, some anatomic anomalies of the ostiomeatal complex (Figure 1) or sphenoethmoidal recess impairing sinonasal drainage can be causative factors mainly in chronic or recurrent disease. The presence of hypertrophic and super-infected adenoidal pads may favour the development of paediatric CRS, acting as an infectious focus.

Children affected by CRS may be candidates to surgery in case of failure of maximal medical therapy including three to six weeks of broad-spectrum systemic antibiotics with adjunctive therapies [8,9,10,11,12,13,14,15]. Although endoscopic sinus surgery (ESS) is the surgical treatment of choice in adult patients with CRS, different surgical procedures are scheduled for refractory paediatric CRS and include adenoidectomy, paediatric ESS (PESS), and balloon catheter sinuplasty (BCS) [8,9,10,11,13,14,15]. In 2012, the European Position Paper on RS and Nasal Polyps designed a therapeutic algorithm for the surgical treatment of paediatric RS, proposing adenoidectomy with possible antral irrigation or maxillary BCS as first-line treatment, and ESS as a second option in the case of failure [3]. 

In the case of complicated disease during acute exacerbations, surgical treatment using ESS and/or an external approach depending on the location should be used in patients with subperiosteal abscess (SPA), orbital abscess or intracranial complications under an emergency setting. Surgery should also be considered if no improvement is observed 24–48 h after parenteral antibiotic treatment, in the case of multiple bacterial infections, and in children aged >9 years or those with known immunodeficiency [16]. In patients with a small SPA without impaired visual acuity or increased intra-ocular pressure, surgery can be considered a second step in the case of worsening of ophthalmological findings or no improvement after 48 h [16].

The present paper provides an overview on surgical options actually available to treat children with CRS. 

## 2. Methods

Pertinent studies published up to 1 February 2019 and concerning the role of surgery in paediatric CRS were selected by ST after a MEDLINE search (accessed via PubMed) based on the following terms: “chronic rhinosinusitis AND children AND surgery”, “chronic rhinosinusitis AND children AND adenoidectomy”, “chronic rhinosinusitis AND children AND surgical treatment”, “chronic rhinosinusitis AND children AND endoscopic sinus surgery”, “chronic rhinosinusitis AND children AND balloon”. The literature searched was aimed to evaluate the effectiveness of any surgical option (i.e., adenoidectomy, BCS, and ESS), as attested by the reported success rate and related failure and complications rates when available.

Consideration was only given to original in vivo studies published in the English language in peer-reviewed journals and specifically concerning the role of surgery in otherwise healthy children with CRS. Animal studies, reviews, and case series including children with systemic disease (cystic fibrosis, primary ciliary dyskinesia, immunological defects) or adult patients were excluded. 

The reference lists were subsequently reviewed to ensure that all of the selected papers were truly relevant and to identify any that had possibly been overlooked.

## 3. Results

Seventeen (including ten prospective non-randomised studies, 6 retrospective studies, and one prospective randomised study) [17,18,19,20,21,22,23,24,25,26,27,28,29,30,31,32,33] of the 72 initially identified papers were included in this review, corresponding to 1930 paediatric patients. 

Success rates range between 47% and 61% after adenoidectomy alone and 87–92% when combined with other surgical procedures. Failures rates were 40–50% and 3–7%, respectively. Success rates after BCS (with or without concomitant procedures) and ESS were 80–100% and 62–87%, respectively, and the corresponding values for failures were 9–19% and 2–12%, respectively. Table 1 shows the main characteristics of the selected case series and related surgical results.

### 3.1. Adenoidectomy

It is well known that adenoids may act as a reservoir for resistant polymicrobial bacterial biofilms responsible for repeated acute exacerbations and recalcitrant disease in children with recurrent upper airway infections, including rhinosinusitis [34,35,36,37,38]. Particularly, Zuliani et al. [38] documented, by means of scanning electron microscopy analysis, that bacterial biofilm was present in almost all the adenoidal specimens taken from children undergoing adenoidectomy for CRS, and it was absent in those specimens removed from children with sleep-disordered breathing but with no history of recurrent infections.

Therefore, adenoidectomy has been proposed as the first-line surgical approach after traditional conservative medical treatment [17,21,39,40,41] in children with symptoms of CRS, being particularly effective in younger ones, with a symptomatic relief reported in up to 80% of cases [1,21,40]. 

A prospective study conducted on 37 children with CRS documented the effectiveness of adenoidectomy in reducing the number of acute exacerbations and in improving the nasal patency, mainly in younger patients with obstructive symptoms [17]. However, it was also reported that a positive surgical outcome was not strictly related to the grade of adenoidal hypertrophy [42,43], and some authors found a good microbiological correspondence between adenoidal bacterial strains and those isolated from the middle meatus [44]. This evidence once again supports biofilm theory, thus suggesting the importance of surgical debridement of any primary biofilm-substained adenoidal infectious focus in children with CRS. 

However, the success rates reported in the literature after adenoidectomy performed for paediatric CRS considerably vary, ranging between 50% and 92% [18,21,23,31,40,41,45,46], based on the case series, different surgical approach (curette vs. endoscopic adenoidectomy), and successful outcomes (mainly expressed as parents reporting symptomatic relief from rhinorrhoea, post-nasal drip, cough, and nasal congestion). It has also been reported that asthmatic children and those younger than seven years old would require more surgery sooner after adenoidectomy [31]. 

Given the possibility of incomplete recovery, some authors resorted to adjuvant procedures, including middle meatal irrigation and maxillary BCS [18,20,27]. Among these, Ramadan et al. [18] reported a better success rate of combined adenoidectomy and maxillary sinusal washes than adenoidectomy alone in children with more severe sinus disease, but not in those with a mild disease. Antral lavage aims both to clear maxillary secretions and collect microbiological samples to guide focused antibiotic treatment. Some studies have found that a therapeutic protocol combining adenoidectomy with antral lavage and long-term intravenous antibiotic treatment was superior to adenoidectomy alone in achieving symptomatic relief [45,47]. BCS may be considered an option too: Ramadan and Terrell [20] documented that adenoidectomy plus balloon catheter sinuplasty was more effective than adenoidectomy alone (especially in older children) in term of symptom improvement 12 months post-operatively.

Taken together, these data suggest that, currently, simple and low-risk adenoidectomy can still be considered a good first-line approach in refractory paediatric CRS possibly combined with sinusal irrigations or BCS, and it should be offered before ESS, especially in younger children without underlying predisposing factors for chronic disease. The main limitations of adenoidectomy are related to patient selection because the procedure seems to be more effective in children younger than 6 years and in non-asthmatic ones. Additionally, the risk of adenoidal regrowth is not negligible. Regarding surgical techniques, based on our experience with endoscopic-assisted adenoidectomy [48], we consider this approach as the preferred modality because it achieves good surgical debridement, with a decreased risk or persistent infectious foci. However, given that no comparative studies about different surgical modalities have been performed, it is not possible to proclaim the superiority of any treatment modality. 

### 3.2. Balloon Catheter Sinuplasty (BCS)

In the last decade, BCS of the maxillary and frontal sinuses has been proposed as a minimally invasive and safe approach for paediatric CRS, allowing the recovery of sinusal ventilation with minimal tissue trauma because it does not require any bone or tissue removal, resulting in minimal post-operative debris and inflammation [49]. It has been reported that the association of BCS with adenoidectomy would enhance the effectiveness of surgery from 50% to 80% [20]. The success rate after BCS ranges between 80 and 100% of cases, according to the case series. In many papers, BCS is performed in combined procedures with adenoidectomy or ethmoidectomy. 

In 2009, Ramadan et al. [26] reported an overall 91% success rate in a cohort of 30 children aged 4–16 years (mean age 8 years) with CRS refractory to medical treatment who underwent balloon catheter sinuplasty combined with sinus washes; 43% of them had concurrent adenoidectomy, while 37% had previously undergone adenoidectomy. No intra- or post-operative complications were reported, but the procedure was not feasible in 9% of cases (including four hypoplastic maxillary sinus cases and one frontal sinus case that was not cannulated) (success rate for non-hypoplastic sinuses: 98%; success rate for hypoplastic sinuses: 60%).

In 2012, Ramadan et al. [27] evaluated the impact of BCS in 26 patients aged 4 to 12 years (mean age; 7.7 years) with symptom recurrence after adenoidectomy: they reported a significant improvement in the mean post-operative Sinus and Nasal Quality of Life Survey score, and surgical success in 81% of cases.

More recently, Soler et al. [28] published a prospective multicentre study to evaluate the effectiveness of BCS in 50 patients (corresponding to 157 treated sinuses, including 98 maxillary, 30 frontal, and 29 sphenoid sinuses) aged 2 to 21 years. In 40% of cases, BSC was the only surgical procedure; however, in the remaining patients, concurrent surgery was performed (adenoidectomy, inferior turbinate reduction, or ethmoidectomy). Dilatation was successfully performed in all the sinuses with no complication, and a significant clinical improvement was attested by an improvement in the Sinus and Nasal Quality of Life Survey score in all the patients at the 6-month follow-up visit.

Thottam et al. [29] retrospectively compared the effectiveness of BCS with ESS (maxillary antrostomy with or without frontal sinusotomy and total ethmoidectomy) and ESS alone (maxillary antrostomy with or without frontal sinusotomy and total ethmoidectomy) in 31 children with a mean age of 9 years: they found no difference in the overall improvement but reported a better outcome in the BCS group in terms of the post-operative use of medications, sinus congestion and headache. On the other hand, Gerber et al. [24] reported, in a prospective randomised trial performed on 25 children, that the addition of BCS to adenoidectomy/maxillary sinus irrigation did not result in additional benefits in terms of the quality of life or symptomatic improvement.

Despite this procedure seeming very attractive, especially in children, given its effectiveness and low morbidity, the radiation exposure risk when radiography is used to confirm balloon placement cannot be neglected, and the presence of peculiar anatomic conditions, particularly hypoplastic sinuses or significant ethmoidal disease, might impair the results [26]. Moreover, because most studies combined BCS with other surgical techniques and prospective randomised trials are missing, it is unclear how much real symptomatic benefit is derived from BCS alone.

### 3.3. Paediatric Endoscopic Sinus Surgery for Chronic Rhinosinusitis

ESS is a safe and commonly used procedure to treat CRS in adult patients, but its use in the paediatric population is less standardised, and most ENT surgeons are reticent in applying it in children. In fact, approximately half of ENT specialists have reported considering adenoidectomy before PESS to treat paediatric CRS [50].

The main concerns are related to the supposed impairment in midfacial growth after surgery that has been suggested by animal studies [39]; however, some prospective studies failed to find a significant effect of PESS on midfacial growth in humans [51,52]. Particularly, anthropometric comparisons between children who had undergone PESS and controls who had received only medical treatment documented the lack of any significant difference among the groups in terms of facial growth 10 years after [52].

Evidence supporting the effectiveness of PESS (mainly comprising maxillary antrostomy and anterior ethmoidectomy) to treat paediatric RS is limited because published studies are mainly retrospective and powered randomised controlled trials are lacking. However, some metanalyses combining results obtained by available studies reported success rates ranging between 62 and 87% [8,9]. A review by Rudnik and Mitchell [53] documented that symptomatic relief after PESS may extend a further six months post-surgically.

However, controversy persist concerning surgical indications [54]. Some authors have suggested that all children with CRS without an immune system defect and who did not respond to both maximal medical treatment and adenoidectomy should be candidates for PESS [23]. Others have indicated that PESS should be proposed in children with documented structural abnormalities (such as septal deviations or spurs, impairment in the patency of the ostiomeatal unit, and nasal polyps) non-responding to maximal medical treatment [55]. Otherwise, other studies have failed to identify specific subgroups of children who are more likely to benefit from PESS after failure of medical treatment [56].

PESS is generally considered a safe procedure. However, but possible untoward effects may infrequently occur, being reported in 0.6–1.4% of cases [8,9]. They include nasal bleeding and major events such as orbital complications, cerebrospinal fluid leak, and meningitis. Additionally, the failure rate cannot be neglected because it has been reported that approximately 13% of otherwise healthy children (i.e., excluding children with cystic fibrosis, immune deficiency, and impaired ciliary activity) who had previously undergone ESS for CRS required revision surgery for adhesion, maxillary sinus ostium stenosis or missed maxillary sinus ostium during primary surgery, or for the development of contralateral disease in the non-operated sinus [26]. Moreover, it was reported that children with asthma and those younger than six years would have a higher risk of revision surgery, suggesting that a more conservative approach would be desirable in such a cohort of patients [26]. Therefore, PESS can be considered an age-dependent procedure, being more effective in older children; it should be performed by experienced surgeons given the well-known risk of rare but severe complications and the more technical complexity of this approach in the paediatric population than in the adult population based on the children’s smaller features.

## 4. Conclusions

Therapeutic management of paediatric CRS requires a stepwise approach with recourse to surgery only after the failure of maximal medical treatment. Surgical options include adenoidectomy, antral washes, BCS, and ESS. Although several studies have been conducted, most were retrospective and uncontrolled, and more surgical procedures were often combined. Moreover, several studies have been published by the same author; thus, it is not possible to exclude some overlap between case series. Therefore, there is an urgent need for randomised controlled studies to assess the clinical benefits derived from single surgical options.

**Figure 2:**
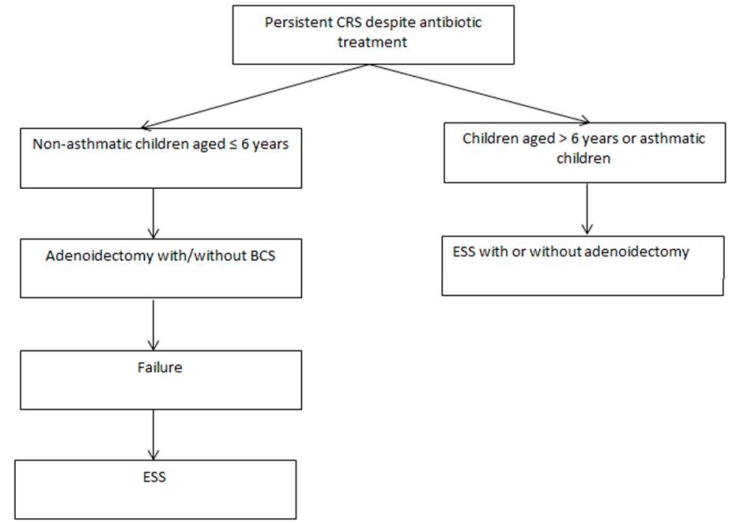
Protocol treatment. CR = chronic rhinosinusitis; BCS = balloon catheter sinuplasty; ESS = endoscopic sinus surgery.

However, given the amount of current evidence, it is reasonable to believe that, in young and otherwise healthy children with refractory CRS, adenoidectomy (eventually combined BCS) should be offered as the first-line surgical treatment. Nevertheless, this approach might be ineffective in some patients who should be candidates for traditional ESS (possibly preceded by BCS if not previously performed). In the case of peculiar conditions (older children with significant ethmoidal disease, hypoplastic sinuses, children with underlying systemic or local predisposing factors), traditional ESS should be considered as the primary treatment. Specific anatomic abnormalities impairing the patency of the ostiomeatal complex should be promptly corrected (Figure 2).

Regardless of the surgical option chosen, some failures may occur, and appropriate pre-operative counselling is desirable to inform the parents of the risk and advantages of each procedure.

## Figures and Tables

**Table 1:** Results of the included studies.

Author, Year	No. of pts, Age (Years)	Study Design	Interventions	Outcomes	Complications/Failures
Ungkanont and Damrongsak [17], 2004	37, Mean: 6.7 ± 2.8	Prospective single arm	Adenoidectomy (±tonsillectomy/myringotomy and tube insertion).	Significant reduction in the number of episodes of acute sinusal infection. Significant reduction in obstructive symptoms.	-
Ramadan and Tiu [18], 2007	121	Retrospective single arm	Adenoidectomy.	Successful in 50% of cases.	Failures in 50% of cases (55 pts. Candidates to ESS, mean age 6.9 years); Failure most frequently in children with asthma and in ≤7 years
Ramadan and Cost [19], 2008	60, Mean: 6.3	Retrospective non-randomised	Adenoidectomy (electrocautery desiccation) with (53%) or without (47%) maxillary sinus wash.	Symptoms * improvement more frequently in children undergone adenoidectomy + sinus wash (87.5% vs. 60.7%). * nasal obstruction/congestion, purulent drainage, cough, headache	ESS performed in two pts for failure
Ramadan and Terrel [20], 2010	49, Mean: 7.7	Retrospective non-randomised	BCS (61%) or adenoidectomy (39%).	Symptoms improvement * more frequently in BCS compared to adenoidectomy (80.0% vs. 52.6%); BCS is more effective in older pts. * SNOT-5	-
Vandenberg and Heatley [21], 1997	44, Range: 1–12	Retrospective single arm	Adenoidectomy.	Complete symptomatic recovery in 58%. Reduction in the number of pts. With rhinorrhoea, nasal congestion, mouth breathing, antibiotic use.	ESS performed in 6.8% for failure
Deckard et al. [22], 2011	110, Mean: 3.7	Retrospective non-randomised	Bilateral maxillary sinus aspiration through the inferior meatus and irrigation (MSI) with adenoidectomy (58.2%) or endoscopically guided middle meatus cultures (ECG) and antral biopsy with adenoidectomy (41.8%).	Recovery in 94.6% of MSI and 92.6% of ECG. Significant reduced time resolution of symptoms in ECG group.	Epistaxis in 1 case (MSI, after trocar introduction) with nasal packing; 2 pseudoproptosis with spontaneous recovery in MSI group
Ramadan [23], 1999	61, Mean: 7.2 ± 3.1	Prospective non-randomised	ESS * (52%) or adenoidectomy (48%). * anterior ethmoidectomy + middle meatal antrostomy; in 28% of cases with posterior ethmoidectomy; in 12% of cases with sphenoidotomy	Symptoms * improvement in 77% of pts. after ESS and in 47% of pts. after adenoidectomy. Improvement in nasal congestion/cough similar in both groups. * nasal congestion, discharge, cough, headache	Adenoidectomy performed in 3% of cases after ESS for failure; ESS performed in 40% of cases after adenoidectomy for failure
Gerber and Kennedy [24], 2018	25, Range: 2–12	Prospective randomised	BCS of the maxillary sinus with antral irrigation and suction electrocautery adenoidectomy (48%) or suction electrocautery adenoidectomy with maxillary antral irrigation via middle on inferior meatus puncture (52%).	Similar improvement in QoL scores and SN-5 * scores in all domains in both groups. * N. of sinus infections, nasal obstruction, allergy symptoms, emotional distress, activity limitations	-
Ramadan et al. [25], 2010	32 *, Mean; 6.5 ± 2.6 * previous adenoidectomy in 56%.	Prospective non-randomised	BCS * with: adenoidectomy (46.9%), anterior ethmoidotomy (15.6%), with antero-posterior ethmoidotomy (3.1%). * 63 sinuses	Successful dilatation in 89% of sinuses * Significant improvement in 50% of cases, partial improvement in 37% of cases. Significant improvement in mean post-operative SN-5. * 94% of maxillary, 67% of frontal, and 57% of sphenoid sinuses	No major complications. Failure in 11% of cases * * 3 hypoplastic maxillary sinuses, 3 sphenoid and 1 frontal sinuses
Ramadan [26], 2009	30 *, Mean: 8, range: 4–16 * previous adenoidectomy in 37%.	Prospective single arm	BCS * (adenoidectomy by means of suction cautery desiccation in 43%). * 56 sinuses: 48 maxillary, 6 sphenoid, and 2 frontal sinuses	Successful dilatation in 91% *. * 98% of non-hypoplastic sinuses, and 60% of hypoplastic sinuses	No major complications. Failure in 9% of cases * * mainly in hypoplastic sinuses
Ramadan et al. [27], 2012	26 *, Mean: 9.0 ± 2.5 * after adenoidectomy failure.	Prospective non-randomised	BCS * (with: anterior ethmoidectomy in 4 pts., contralateral maxillary antrostomy for hypoplastic sinus or failure to cannulate in 3 pts., revision of adenoidectomy in 2 pts.). * 33 sinuses	Success in 81% of cases. Significant improvement in post-operative SN-5.	Failure in 19% of cases.
Soler et al. [28], 2007	50	Prospective single arm	BCS * (combined procedures in 60% including adenoidectomy in 42%, inferior turbinate reduction in 26%, ethmoidectomy in 12%). * 157 sinuses: 98 maxillary, 30 frontal, 29 sphenoid sinuses.	Success in 100% of cases. Significant improvement in 94% of cases. Significant improvement in post-operative SN-5.	Minor side effects in 2 pts. No improvement in 6% of cases. No revision surgery performed.
Thottam et al. [29], 2012	31 *, Mean: 9.3, range: 3–17 * previous adenoidectomy in 13 pts.	Retrospective non-randomised	BCS * with ethmoidectomy (15 pts.) or ESS ** (16 pts.). * 30 maxillary and 10 frontal sinuses. ** maxillary antrostomy in 32 sinuses + DRAF I/IIA frontal sinusotomy in 12 frontal sinuses.	Improvement in sinus complaints * in 62.5% of pts. after ESS and in 80% after BCS. Significant improvement of congestion in BCS groups compared to ESS group. * facial pain, congestion, post-nasal drip, rhinorrhoea, headache, nasal spray use.	No complications. ESS performed in 1 pt. after BCS for failure. No improvement/worsening in 3 pts. of BCS group and in 6% of ESS group.
Ramadan [30], 2001	83 *, Mean: 5.98, range: 2–14. * previous adenoidectomy in 26 pts.	Prospective non-randomised	ESS (anterior ethmoidectomy + maxillary antrostomy in 112 pts., posterior ethmoidectomy + sphenoidotomy in few cases) with adenoidectomy (62%).	Success in 82%. Significantly higher success rate in children ≥6 years.	Revision surgery in 11 of cases (mainly in younger than 6 years).
Ramadan [31], 2004	202, Range: 2–13	Prospective non-randomised	ESS with adenoidectomy * or ESS or adenoidectomy * * curetting and/or cauterisation	Success in 87.3% of pts. after ESS with adenoidectomy, in 75.0% after ESS, and in 51.6% after adenoidectomy. Best results after ESS with adenoidectomy, followed by ESS.	Minor orbital complications * in 2.9% of cases. Revision surgery in 7.6% of pts. after ESS with adenoidectomy, in 12.5 after ESS, and in 51.6% after adenoidectomy. * orbital entry and ecchymosis.
Jiang et al. [32], 2006	729, Range: 12–18	Prospective single-arm	ESS	Symptomatic improvement at post-operative SNOT-20	-
Ramadan and Hinerman [33], 2006	141	Prospective single-arm	ESS	Success in 80% of pts.	-

ESS: endoscopic sinus surgery; N: number; Pts: patients, BCS: balloon catheter sinuplasty; QoL: quality of life.
